# Evaluation of Functional Outcomes After Reconstruction of Post-burn Contracture of the Hand in the Pediatric Age Group: A Prospective Study

**DOI:** 10.7759/cureus.57102

**Published:** 2024-03-28

**Authors:** Shobhana Rajasekar, Karthikeyan Gomathinayagam, Sriman Narayan Madan Mohan

**Affiliations:** 1 Department of Hand Surgery, Sri Ramachandra Institute of Higher Education and Research, Chennai, IND; 2 Department of Plastic and Reconstructive Surgery, Tamil Nadu Government Multi Super Speciality Hospital, Chennai, IND; 3 Department of Orthopaedics, Sri Ramachandra Institute of Higher Education and Research, Chennai, IND

**Keywords:** good functional outcome, pediatric age group, modified jebson hand function test, reconstructive surgical procedure, post-burn contracture

## Abstract

Background

Injuries on the hand due to burns affect the quality and functions of activities of daily living (ADL). Severe burns cause lasting complications and deformities, such as contracture of the hand, which require multiple staged surgeries and rehabilitation for extended periods to regain function. This is of major significance to children, as they are in the growing and developmental age group, which should be considered while planning a reconstructive procedure. Psychological counselling is equally important for these patients to accept the residual deformity and cosmesis of the hand following surgery.

Methods

A prospective interventional study was conducted on 40 patients to assess the functional outcomes of various reconstructive procedures for post-burn contracture of the hand and to find out which is the better surgical intervention for restoring the hand functions needed for ADL. Functional outcomes were analyzed using the Modified Jebson Hand Function Test (JHFT) after a minimum of four months.

Results

In the group of children operated on with flap procedures, the maximum number of patients had average functional outcomes. Functional outcomes were assessed using the Modified JHFT, in which fine motor, weighted, and non-weighted hand function activities were assessed and analyzed. However, in the group of children operated on by the Z-plasty procedure and skin graft procedure, the maximum number of patients had poor functional outcomes.

Conclusion

The management of burn injuries on the hand and subsequent contractures is often challenging, especially in pediatric patients. Timely intervention, patient education, and surgical skills with an appropriate choice of reconstructive procedures play a vital role in achieving good postoperative results. This study showed that hands reconstructed using flap procedures had good functional outcomes compared to graft and Z-plasty procedures.

## Introduction

Children sustaining injuries due to burns is a major epidemiologic problem around the globe. Nearly a fourth of all burn injuries occur in children under the age of 16, of whom the majority are under the age of five [1]. Children are commonly affected by burn injuries, particularly scalds due to spillage of hot liquids, and the part of the body that is most commonly affected is the hand [2]. Failure to seek timely medical intervention, inadequate medical care, and lack of care during healing and rehabilitation are common causes of post-burn hand contracture. Of the various causes of hand contractures, the most common cause is injuries from burns [3]. This is due to the intricate anatomy of the hand, which consists of thin dorsal skin, a rich sensory supply of palmar skin, and the intrinsic musculature of the hand, along with tendons that suffer extensive damage due to burn injuries. Children have a relatively thinner dermis, so any given thermal insult will sustain a deeper burn than an adult [4]. A burn injury can impair skin integrity and sensation and lead to hypertrophic scarring. The activities of daily living (ADL) are greatly hampered due to scarring and contracture that affect the hand function following burns. There is also severe compromise in the aesthetic appearance of the hand. The appropriate choice as well as the timing of surgery with physiotherapy and rehabilitation are extremely important to improve the functional outcome for a survivor of a burn injury.

A variety of reconstructive procedures have been employed for the treatment of post-burn hand deformities, which include Z-plasty and local flaps, regional flaps, island flaps, free flaps, and skin grafting, with each technique having its advantages and disadvantages. It is of paramount importance to assess the efficacy of different procedures as far as functional recovery and aesthetic improvement are concerned. This study was performed to assess the outcome of the surgical reconstructive procedures and to ascertain which intervention is better to restore the hand functions needed for ADL.

## Materials and methods

This was a prospective interventional study conducted in the Department of Burns, Plastics, and Reconstructive Surgery, Government Kilpauk Medical College Hospital, Chennai, India, following approval from the institutional ethics committee of Government Kilpauk Medical College (approval ID no. 22/2017) for three years between January 2014 and January 2017. The study aimed to analyze the functional outcomes of the reconstructive procedures performed for the correction of post-burn hand contracture and to assess the broad range of hand functions required for ADL that are restored after each type of reconstructive procedure. The inclusion criteria included patients with post-burn contractures of the hand operated on between January 2014 and January 2017 in the age group of three to 18 years who had interference with daily activities of life. Only children whose dominant hand was involved were included in the study. The exclusion criteria encompassed patients with hand contractures with raw areas and unstable scars, patients with electrical burns, and contractures of McCauley severity of Grades 1 and 2 [5].

All children who came to the hospital with post-burn hand contracture, from age groups of three to 18 years of age, from January 2014 to January 2017, were assessed clinically for site and type of contracture, its effect on daily activities of life, and classified according to McCauley severity classification into four grades of severity [5].

The sample size was 40 cases. The sample size was calculated based on the reference article “Study on Surgical Management of Post-burn Hand Deformities,” which was authored by Nonavinakere Sunil et al. in the Journal of Clinical and Diagnostic Research [3]. In this study, 75% of cases had a good recovery after reconstructive surgery for post-burn contracture. The sample size was calculated using OpenEpi software (The OpenEpi Project, Atlanta, GA). The description is that when the percentage with a good outcome is 75, the power of the study is 80%, and the confidence level is 95%, to pick up a difference of 45 between groups, 40 is the minimum sample size required.

Patients with Grades 1 and 2 post-burn hand contractures were managed by non-surgical approaches, such as physiotherapy and scar control measures, and were thus excluded from the study. Patients with Grade 3 and Grade 4 post-burn scar contracture (PBSC) were included in the study group. These contractures affected the daily activities of life of the children. The post-burn contracture of the hands was reconstructed by either Z-plasty, graft, or flap procedures depending upon the site and shape of the contractures, the size of the raw area, and the structures exposed following contracture release.

Postoperatively, active physiotherapy was started as soon as the surgical wounds healed. Scar control measures such as pressure massage, silicone sheets, and pressure garments were started after three weeks, once the healing of the graft or flap was ensured. Patients were followed up weekly for two weeks, bi-weekly for one month, and every month after that. Functional outcome was analyzed using the Modified Jebson Hand Function Test (JHFT) after a minimum of four months. With this test, fine motor, weighted, and non-weighted hand function activities were assessed. Sensory assessment was out of the purview of this study. The statistical analysis for the data obtained from the study was done using Pearson's chi-square test, one-way analysis of variance test (ANOVA), and post hoc test.

## Results

Out of 40 patients in the study group, only four were left-dominant, and the remaining 36 were right-dominant. The youngest patient was three years old, and the oldest was 17 at the time of surgery in the study group, with a mean age of 9.4 years. Of the 40 patients in the study group, 21 (52%) and 19 (48%) were female and male, respectively.

In our study group of 40 hand contractures, the volar surface of the fingers was involved in 28 cases, the dorsum of the hand was involved in five cases, and the palm was involved in four cases of hand contractures. Other sites of contractures included the dorsal surface of fingers, which was involved in two cases of hand contracture, and the first webspace, which was involved in two cases of hand contracture. One case of contracture involved the volar surface of the wrist; one case involved the dorsum of the wrist; one case involved the volar surface of the thumb; and one case of contracture involved the dorsal surface of the thumb. The third and fourth web spaces were involved in one case of hand contracture. Out of 40 cases in our study group, six cases had concomitant contractures.

**Figure 1:**
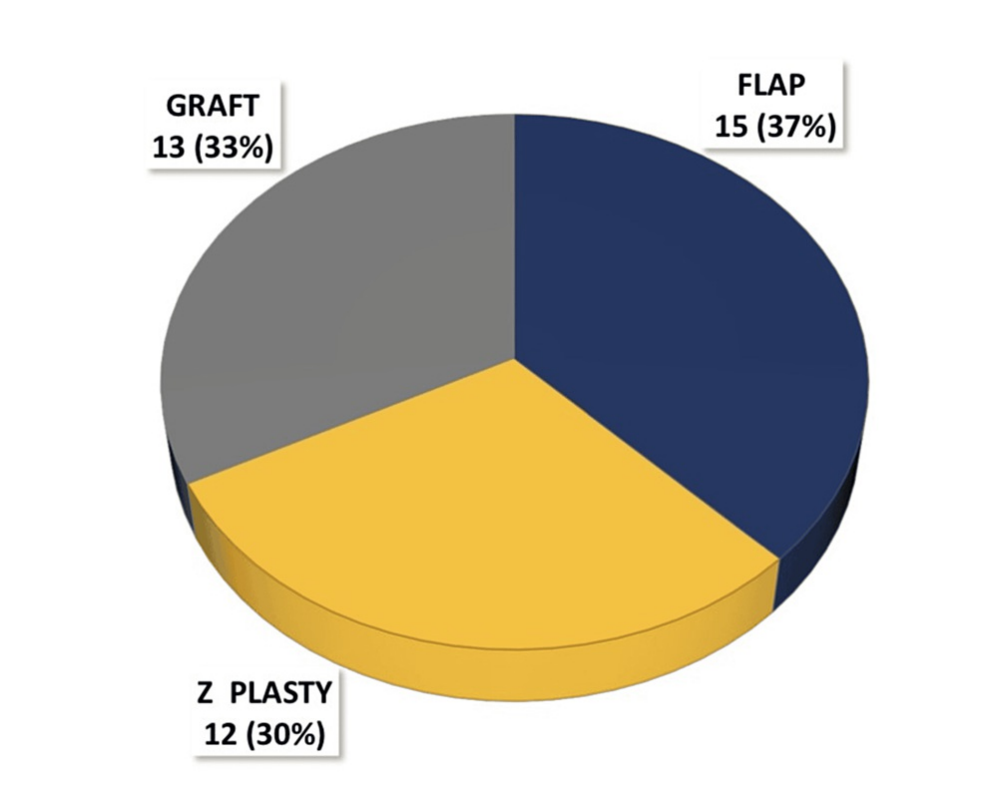
Distribution of patients according to graft, flap, and Z-plasty procedures

Of these patients, 13 underwent a graft procedure, 12 underwent a Z-plasty procedure, and 15 underwent a flap procedure (Figure 1).

**Figure 2:**
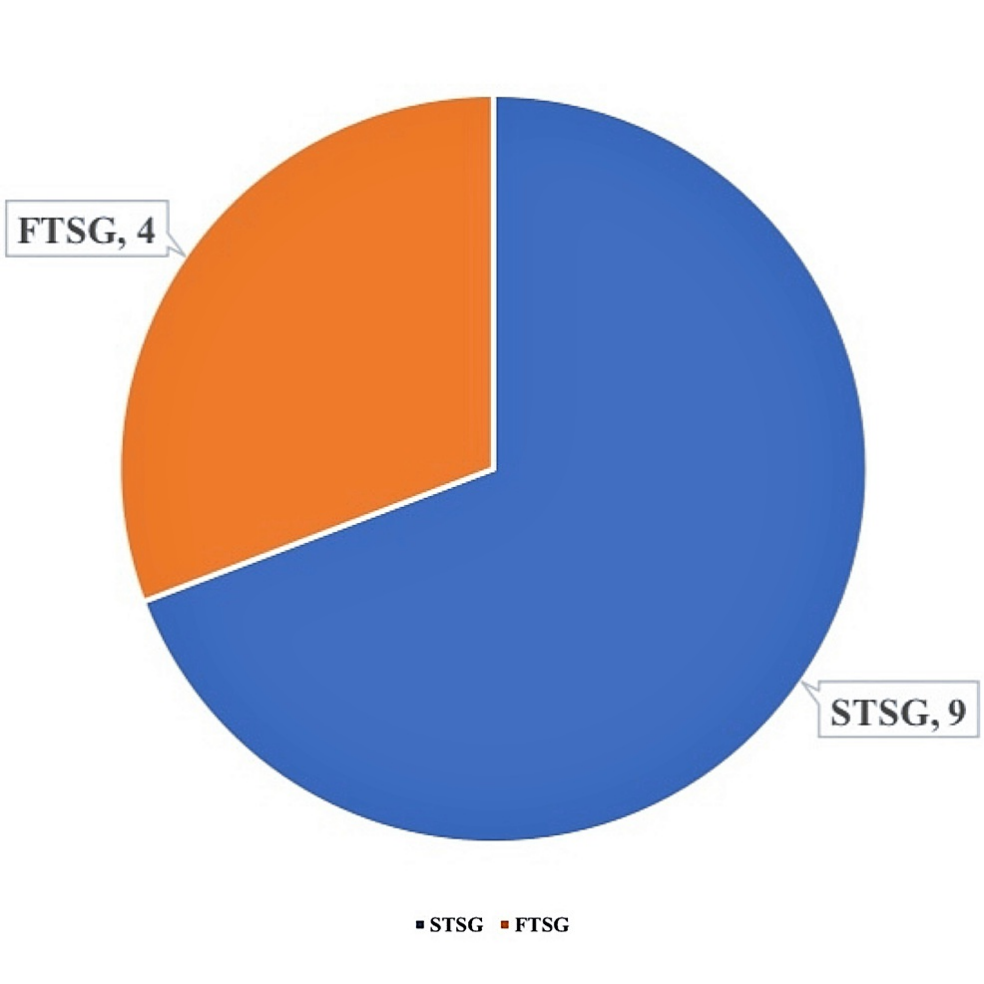
Distribution of patients who underwent split-thickness skin grafting (STSG) and full-thickness skin grafting (FTSG)

Of the 13 graft procedures performed, split-thickness skin grafting (STSG) was done for nine patients, and full-thickness skin grafting (FTSG) was done for four patients (Figure 2).

**Figure 3:**
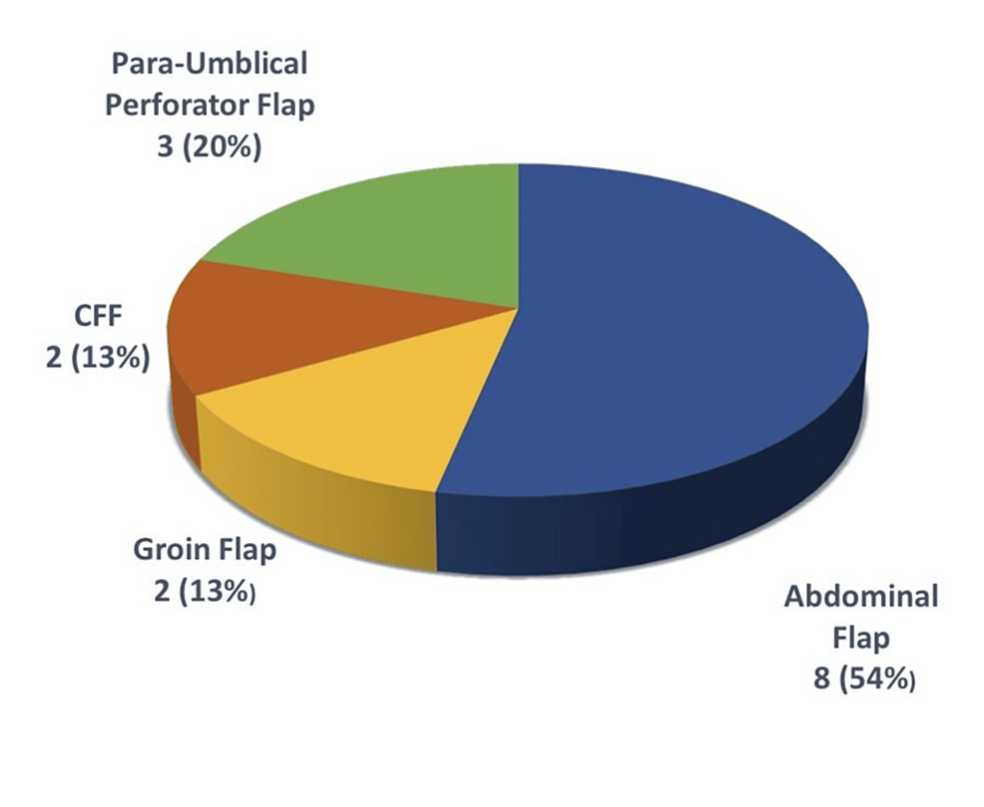
Distribution of patients who underwent para-umbilical flap, cross-finger flap (CFF), groin flap, and abdominal flap procedures

Of the 15 flap procedures performed, eight patients underwent a superiorly/inferiorly based abdominal flap, two patients underwent a groin flap, two patients underwent a cross-finger flap (CFF), and three patients underwent a para-umbilical perforator flap (Figure 3).

All patients were assessed after a minimum follow-up period of four months after surgery.

In 25 patients, the time interval of assessment was four to six months, and in 15 patients, the time interval of assessment was six months to one year. The functional outcome was assessed using the Modified JHFT. The JHFT is an assessment tool used to analyze the outcomes of various reconstructive procedures in the pediatric population [6, 7]. It consists of seven subtests (activities) that include a range of fine motor, weighted, and non-weighted hand function activities that are timed. The subtest score was the time (in seconds) taken to complete each subtest, and the total score was the sum of the time taken to complete each subtest (in seconds). The lower score indicates greater hand function.

In this study, the modification was done in the first subtest of the originally described JHFT, as it was difficult for a three-year-old child to perform it and was simplified by asking them to draw simple pre-set depicted patterns. The rest of the six subtests are the same as those of the JHFT.

The results of 13 cases reconstructed with the graft procedure using the Modified JHFT are shown in Table 1.

**Table 1:** Results of the cases reconstructed with the graft procedure using the Modified Jebson Hand Function Test Each column represents the time taken to complete each activity in seconds, and the last column represents the total time taken to complete all seven activities in seconds. lb: pound

Patients who underwent the graft procedure	Drawing preset depicted patterns	Card turning	Picking small common objects & placing them in a container	Stacking checkers	Stimulated feeding	Moving light objects (empty can)	Moving heavy objects (1 lb weighted can)	Total score (Total time taken in seconds)
1	37	4	3	23	3	4	6	80
2	45	6	5	30	3	3	4	96
3	50	6	5	32	3	2	4	102
4	49	5	5	33	4	2	3	101
5	43	4	4	31	3	3	4	92
6	58	7	5	35	3	3	4	115
7	38	5	3	25	3	2	5	81
8	37	5	4	26	3	4	6	85
9	50	6	5	30	3	2	4	100
10	46	6	5	32	3	2	4	98
11	45	7	5	35	3	2	3	100
12	40	3	5	31	4	3	5	91
13	43	6	5	35	3	2	4	98

Table 2 shows the results of 12 cases reconstructed with the Z-plasty procedure using the Modified JHFT.

**Table 2:** The results of the cases reconstructed with the Z-plasty procedure using the Modified Jebson Hand Function Test Each column represents the time taken to complete each activity in seconds, and the last column represents the total time taken to complete all seven activities in seconds. lb: pound

Patients who underwent the Z-plasty procedure	Drawing preset depicted patterns	Card turning	Picking small common objects & placing them in a container	Stacking checkers	Stimulated feeding	Moving light objects (empty can)	Moving heavy objects (1 lb weighted can)	Total score (Total time taken in seconds)
1	56	6	5	32	3	2	3	107
2	48	5	4	34	4	3	3	101
3	45	6	5	30	3	2	4	95
4	49	6	5	36	4	4	6	110
5	44	6	5	32	4	2	5	98
6	43	6	5	30	3	3	4	94
7	21	2	3	22	2	2	4	56
8	25	3	4	26	3	4	5	70
9	38	4	4	27	3	2	5	83
10	39	4	4	35	3	3	4	92
11	25	3	4	24	3	2	4	65
12	43	6	5	35	3	2	4	98

Table 3 shows the results in 15 cases reconstructed with the flap procedure using the Modified JHFT.

**Table 3:** The results of the cases reconstructed with the flap procedures using the Modified Jebson Hand Function Test Each column represents the time taken to complete each activity in seconds, and the last column represents the total time taken to complete all seven activities in seconds. lb: pound

Patients who underwent the flap procedure	Drawing preset depicted patterns	Card turning	Picking small common objects & placing them in a container	Stacking checkers	Stimulated feeding	Moving light objects (empty can)	Moving heavy objects (1 lb weighted can)	Total score (Total time taken in seconds)
1	46	6	5	29	3	2	4	95
2	38	5	3	24	4	2	5	81
3	42	3	5	30	4	3	4	91
4	36	5	4	26	3	4	6	84
5	39	5	5	30	3	2	4	88
6	27	3	5	28	4	4	5	76
7	35	5	4	30	4	3	5	86
8	25	3	4	26	3	4	5	70
9	23	3	4	24	3	3	5	65
10	35	5	4	29	5	3	4	85
11	22	2	2	20	3	2	3	54
12	37	5	4	26	3	4	6	85
13	38	5	3	25	3	2	5	81
14	38	5	3	25	4	4	6	85
15	37	4	3	23	3	4	6	80

The lowest total score was 54, and the maximum total score was 115.

The total score was divided into three categories, and the outcome was graded as a good outcome if the total score was less than 60 seconds, an average outcome if the total score was between 60 and 90 seconds, and a poor outcome if the total score was more than 90 to 110 seconds.

Table 4 depicts the final results of all 40 patients who underwent reconstructive procedures for post-burn hand contractures.

**Table 4:** Final outcomes of all 40 patients who underwent the various reconstructive procedures

Intervention name	Number of cases as per outcome			Total number of cases
	<60 sec (Good)	60 – 90 sec (Average)	>90 – 120 sec (Poor)	
1. Flap	1	12	2	15
2. Z-plasty	1	3	8	12
3. Graft	0	3	10	13

Of the 15 patients who underwent flap surgery, one patient had a good result according to the Modified JHFT. This patient had post-burn hand contracture involving only the little finger and was reconstructed using a para-umbilical perforator. Twelve patients had average results, and two patients had poor results.

Out of 12 patients who underwent Z-plasty, one had a good result. This patient had a post-burn hand contracture involving the volar side of only the little finger. Three patients had an average result, and the maximum number of patients (eight patients) had poor results. Out of 13 patients who underwent graft surgery, four cases were recipients of FTSG, and nine cases were recipients of STSG. No patient had good results. Out of the four patients who underwent the procedure of FTAG, one patient had an average result. In this patient, the volar side of the index finger was involved. Three patients had poor results, in whom the volar side of multiple fingers was involved. Out of the nine patients who underwent the procedure of STSG, two had average results where only the dorsum hand was involved. Seven patients had poor results. The graft cases gave the maximum total score of 115.

Analysis

Patients whose hands were reconstructed with skin grafts had an average of 1.2 surgeries; those with Z-plasty had an average of one surgery; and those with flap procedures had an average of three surgeries.

The total time needed for reconstruction on average using the split skin graft (SSG) procedure is two months; the Z-plasty procedure is one month; and the flap procedure is 3.5 months. The statistical analysis of the data obtained from the study was done using Pearson's chi-square test, one-way ANOVA, and post hoc test.

Using Pearson's chi-square test, the total time taken to perform all seven activities between 60 and 90 seconds was found in 80% (12) of cases among the flap surgery group, whereas it was found in 25% (3) of cases among the Z-plasty group and 23.1% (3) of cases among the graft surgery group, and this difference was found to be statistically significant (p-value = 0.007) (Table 5).

**Table 5:** Statistical analysis using the chi-square test to determine the association between the total time taken to complete all seven activities and the type of reconstructive surgery.

Type of surgery		Total duration (In seconds)			Total number of cases	Pearson's chi-square test value	p-value
		< 60	60 - 90	> 90 -120			
	1. Flap	1 (6.7%)	12 (80%)	2 (13.3%)	15 (37.5%)	14.123	0.007**
	2. Z-plasty	1 (8.3%)	3 (25%)	8 (66.7%)	12 (30%)		
	3. Graft	0 (0%)	3 (23.1%)	10 (76.9%)	13 (32.5%)		
Total		2(5%)	18 (45%)	20 (50%)	40		
* Significant at p <0.05. ** Very significant at p <0.01.							

The mean time (in seconds) taken to perform activities post-surgery like drawing pre-set depicted patterns, card turning, picking small common objects and placing them in containers, and stacking checkers was found to be less among those cases who underwent flap surgery (Table 6).

**Table 6:** Mean duration of time taken to perform various post-surgical activities lb: pound

Variable	Type of surgery	Total number of cases (N=40)	Mean (time in seconds)	Standard deviation
Drawing preset depicted patterns	1. Flap	15	35	7.0
	2. Z-plasty	12	40	10.8
	3. Graft	13	45	6.1
Card turning	1. Flap	15	4	1.2
	2. Z-plasty	12	5	1.5
	3. Graft	13	5	1.2
Picking small common objects and placing them in a container	1. Flap	15	4	.9
	2. Z-plasty	12	4	.7
	3. Graft	13	5	.8
Stacking checkers	1. Flap	15	26	3.0
	2. Z-plasty	12	30	4.4
	3. Graft	13	31	3.9
Stimulated feeding	1. Flap	15	3	.6
	2. Z-plasty	12	3	.6
	3. Graft	13	3	.4
Moving light objects (empty can)	1. Flap	15	3	.9
	2. Z-plasty	12	2	.8
	3. Graft	13	2	.8
Moving heavy objects (1lb weighted can)	1. Flap	15	5	.9
	2. Z-plasty	12	4	.9
	3. Graft	13	4	.9

The one-way ANOVA test was run, and it was found that the difference in the mean duration of time (in seconds) taken to perform activities post surgery was found to be statistically significant for activities like drawing preset depicted patterns (p = 0.008) and stacking checkers (p = 0.009) (Table 7).

**Table 7:** Statistical analysis using one-way analysis of variance to compare the mean duration of time to perform activities post surgery among three types of reconstructive surgeries lbs: pound

Type of activity		Sum of squares	df	Mean square	F test value	p-value
Drawing preset depicted patterns	Between groups	721.606	2	360.803	5.489	0.008
	Within groups	2432.169	37	65.734		
	Total	3153.775	39			
Card turning	Between groups	8.715	2	4.357	2.675	0.082
	Within groups	60.260	37	1.629		
	Total	68.975	39			
Picking small common objects and placing them in a container	Between groups	3.619	2	1.810	2.804	0.073
	Within groups	23.881	37	.645		
	Total	27.500	39			
Stacking checkers	Between groups	151.365	2	75.682	5.423	0.009
	Within groups	516.410	37	13.957		
	Total	667.775	39			
Stimulated feeding	Between groups	.883	2	.441	1.472	0.243
	Within groups	11.092	37	.300		
	Total	11.975	39			
Moving light objects (empty can)	Between groups	1.723	2	.862	1.292	0.287
	Within groups	24.677	37	.667		
	Total	26.400	39			
Moving heavy object (1lb weighted can)	Between groups	3.247	2	1.624	1.954	0.156
	Within groups	30.753	37	.831		
	Total	34.000	39			

A post hoc test was also run, which showed that the mean difference in time taken to perform activities like drawing pre-set depicted patterns and stacking checkers was found to be significant with flap surgery when compared with Z-plasty and graft procedures (Table 8).

**Table 8:** Comparison among three types of reconstructive surgeries using a post hoc test (multiple comparisons) for the time taken to perform the two activities among patients.

Dependent variable	(I) Type of surgery	(J) Type of surgery	Mean difference (I-J) (time in seconds)	Std. error	p-value	95% confidence interval	
						Lower bound	Upper bound
Drawing preset depicted patterns	Flap	Z-plasty	-5.3	3.1	.300	-13.2	2.6
		Graft	-10.1^*^	3.1	.006	-17.9	-2.5
	Z-plasty	Flap	5.3	3.1	.300	-2.6	13.2
	Graft	-4.9	3.2	.429	-13	3.3
	Graft	Flap	10.2^*^	3.1	.006	2.5	17.9
		Z-plasty	4.9	3.2	.429	-3.3	13
Stacking checkers	Flap	Z-plasty	-3.7^*^	1.4	.047	-7.3	-.0
		Graft	-4.3^*^	1.4	.014	-7.8	-.7
	Z-plasty	Flap	3.7^*^	1.4	.047	.0	7.2
	Graft	-.6	1.5	1.000	-4.4	3.1
	Graft	Flap	4.3^*^	1.4	.014	.7	7.8
		Z-plasty	.6	1.5	1.000	-3.1	4.4
* The mean difference is significant at the 0.05 level. ** The mean difference is very significant at the 0.01 level.							

From the various tools employed, we analyzed that the maximum number of patients with hands reconstructed by flap procedures took less time to complete tasks compared to hands reconstructed with grafts and Z-plasty. We also analyzed drawing pre-set depicted patterns and stacking checkers, which were tests that showed flap procedures gave better hand function after the reconstructive surgery, particularly in terms of fine motor function and precision demands of the hand. In addition to these, we analyzed that even though the number of surgeries and total time needed for reconstruction were less with Z-plasty and graft procedures, they only helped to downgrade the severity of the contracture. i.e., from Grade 3 or 4 to Grade 1 or 2, was less effective in restoring the hand functions needed for ADL.

## Discussion

Post-burn contractures that are severe pose a significant problem in developing countries [8]. Management of burn injuries and their associated complications and post-burn sequelae such as contractures and deformities are of particular significance in developing countries due to various factors such as delayed presentation, non-compliance with protracted follow-up and treatment necessary along with hand physiotherapy and rehabilitation to regain function, as well as causes such as high financial costs associated with reconstructive procedures, the requirement of multiple staged surgeries, and extended duration of treatment. A loss of the hand contributes up to a 57% decline in function for a person, though the hands contribute to less than 5% of the total body surface area [9, 10]. Hands are involved in more than 80% of all severe burns [11]. Even isolated burn injuries to the hand cause significant cosmetic deformity and major functional impairment. It is much more complicated in the pediatric age group as the hand is developing and the burns affect the growth of the hand [12]. The patient's ability to do work following recovery is to a great extent influenced and determined by the residual hand function, and thus treatment of the hands receives high priority as severe hand burns may even disable patients for adequate self-care [13].

Grades of burn scar contracture have been classified, according to McCauley, into four severity grades: Grade 1: symptomatic tightness but no limitation in range of motion, normal architecture; Grade 2: mild decrease in range of motion without significant impact on ADL, no distortion of normal architecture; Grade 3: functional deficit noted, with early changes in the normal architecture of the hand; Grade 4: loss of hand function with significant distortion of the normal architecture of the hand [5]. Splinting of the hand in an anti-deformity position is vital during the time of primary clinical evaluation and treatment. The role of a hand therapist in starting a supervised passive motion protocol promptly following an injury is indispensable. This is also essential within the first two weeks of surgery for the patients to get better functional outcomes. Pressure garments and silicone sheets also play a role in decreasing scar formation by the pressure effect and producing relative tissue hypoxia [14]. Non-surgical treatments are not of much use in reversing the scarring process once functionally limiting contractures have already developed. Therefore, initial management of the acutely burned hand to prevent these complications has rightfully been of concern, and a wealth of literature has accumulated relating to this subject [12].

Lack of adequate primary management leads to post-burn scarring and, subsequently, contractures [8]. The classic rule of burn surgery is to delay secondary procedures until scars have fully matured, which takes approximately one year. The severity of disability and morbidity for each patient depends upon the functional requirements of the hand as per the occupation of the patient as well as the aesthetic and personal requirements. In patients with a purely hypertrophic scar that does not impair the function of the hand by contracture, the surgical excision of the scar may be delayed until the maturation of the scar occurs. However, in patients with severe skin contracture with limitation of motion, intervention by surgery is required before the scar has fully matured to prevent secondary tendon and joint contractures. A thorough evaluation of the deformity should be made while preparing the treatment plan and concentrating on restoring hand function to its maximum. Pre-existing literature suggests that good functional and aesthetic results can be expected from first-degree and superficial second-degree burns, as these injuries have good potential to heal adequately within about two weeks. Deep second-degree (partial skin thickness), third-degree (full skin thickness), and fourth-degree (tendon, bone, nerve, or joint involvement) burns heal by scar tissue formation and so require a longer time to heal [10].

When a hand is severely involved, the choice of the first set of procedures is very important, as it contributes the most to improving the functional outcome of the surgery for the patient [15]. Excision of the scar tissue and correction of the deforming forces, followed by soft tissue reconstruction, are needed for the amelioration of the deformity [16]. Good preoperative planning for reconstruction and opting for the most simple method is needed to achieve the reconstructive goals [17]. Studies by Bunyan and Mathur [18] and Tanabe et al. [19] suggest that FTSGs give a more durable and cosmetically pleasing result than STSGs and are less likely to contract, but the limited availability of FTSGs restricts their usage for larger burns.

Employment of local and regional flaps for the reconstruction of a burnt hand curtails the risk of recurrence [20]. Free flaps, such as the medial plantar artery flap, have been advocated for the reconstruction of palmar defects in burned hands [21-23]. Employing the techniques of abdominal and groin flaps is a good choice for reconstruction of hand defects, but has the disadvantage of prolonged immobilization [24]. The selection of an ideal flap cover from the various options described should be made judiciously, considering factors such as the availability of the donor area, the expected enhancement of the functional outcome of the hand after the surgery, the surgeon’s skill, and most importantly, the need for future reconstructive procedures that may be required [25].

This study had a few limitations. First, the sensory assessment of the patients was not included and was out of the purview of this study. Second, the analysis was done for a wide pediatric age group of three to 18 years. The analysis of subset of age groups was not done, as by doing so, the statistical analysis was getting complicated with too many numericals and did not give any conclusive data or results.

## Conclusions

From the study, we concluded that hands reconstructed using flap procedures had better functional outcomes when compared to skin graft and Z-plasty procedures. The first set of reconstructive procedures is critical, as it provides maximum benefit to the patient in restoring hand function. This is of much significance to children, as they are in the growing and developing age group, which should be considered while planning for a reconstructive procedure. Psychological and physical adjustment to residual deformity may affect the subsequent choice of lifestyle and occupation of the patient. In addition to these factors, support from the community and family, along with extensive hand therapy for rehabilitation, plays a pivotal role in encouraging the patient to continue further follow-up and improve hand function. Timely intervention, patient education, and surgical skills with an appropriate choice of reconstructive procedures play a vital role in achieving good postoperative results.
